# Scalable Surface Microstructuring by a Fiber Laser for Controlled Nucleate Boiling Performance of High- and Low-Surface-Tension Fluids

**DOI:** 10.1038/s41598-018-25843-5

**Published:** 2018-05-10

**Authors:** Peter Gregorčič, Matevž Zupančič, Iztok Golobič

**Affiliations:** University of Ljubljana, Faculty of Mechanical Engineering, Aškerčeva 6, 1000 Ljubljana, Slovenia

## Abstract

Nucleate boiling enables effective cooling and heat transfer at low temperature differences between a heated surface and the surrounding fluid. It is utilized in many applications, ranging from large power plants to small microelectronics. To enhance the boiling process by minimization of the surface temperature and increase the maximum attainable heat flux, several approaches for surface modifications were recently developed. However, each of them has at least one important drawback, including challenging and expensive production, mechanical and/or thermal instability or problematic scale-up. Herein, a straightforward, robust and flexible method using a nanosecond fiber laser for production of surfaces with multi-scale micro-cavities (with diameters ranging from 0.2 to 10 μm) is developed. Examination of these surfaces in two very contrasting fluids - water, which is polar, has high surface tension and high latent heat of vaporization; and non-polar, dielectric tetradecafluorohexane (FC-72) with low surface tension and much lower latent heat - confirms that such surfaces enable enhanced heat transfer and controlled boiling in combination with diverse fluids. This demonstration suggests that the developed method has the potential to overcome the current limitations for further miniaturization of microelectronic devices and to increase performance and safety in high heat flux systems.

## Introduction

Nucleate boiling is a part of our everyday life as it is one of the most effective heat transfer mechanisms taking place at low temperature differences between the heated surface and the surrounding fluid. It is utilized for cooling and general heat transfer in many applications on various scales - from large nuclear power plants to small microelectronics. On the one hand, the boiling water reactors – the second most common form of nuclear reactors^1^ – use water, i.e., a polar liquid with high surface tension as a working fluid. On the other hand, the dielectric, non-polar fluids with almost one order of magnitude lower surface tension are used for phase-change heat transfer and are already being utilized in heat pipes, which are produced in the hundreds of millions annually and are employed in computers, mobile phones, solar collectors, space applications and other fields^2,3^. Miniaturization of these microelectronic devices is limited by the cooling capabilities, while the future development of high heat flux systems in terms of performance and safety concerns depends on the enhancements of phase-change heat transfer.

In the last century, nucleate pool boiling became the primary field of research in liquid-vapour heat transfer area^4,5^, and the researchers have been trying to enhance the boiling process in order to minimize the surface temperature (e.g., increase the heat transfer coefficient) and increase the critical heat flux (CHF), which defines the maximum attainable heat flux in the nucleate boiling regime^6–11^. Providing a low onset of nucleate boiling (ONB) temperature, creating a high density of active nucleation sites and limiting the bubble growth will increase heat transfer coefficient at low heat fluxes and will also improve the boiling performance at higher heat fluxes^12^. For further enhancement of the CHF, different authors report best results on surfaces, which provide separate liquid-vapour pathways and improve the rewetting of dry spots by means of increased capillary wicking effects^13–16^. When water is used as a working fluid, it is possible to simultaneously decrease the onset of nucleate boiling temperature, increase the CHF and define locations of active nucleation sites by creating hydrophobic/hydrophilic (e.g., biphilic) surface patterns^12,17–19^. However, sole modification of the surface wettability is not the ultimate enhancement approach, since dielectric and non-polar fluids with small capillarity lengths are also used in boiling applications; the effects of the aforementioned surface modifications are greatly reduced in combination with such fluids.

Current surface modification methods for phase-change heat transfer improvement mostly include mechanical machining, chemical treatments (oxidation, chemical vapour deposition), (nanoparticle) coatings, and micro-/nanoelectromechanical systems techniques, such as photolithography and reactive ion etching^20–24^. Each of these methods has one or more important drawback which usually include challenging and expensive production, mechanical and/or thermal instability of surfaces, problematic scale-up and difficult implementation on real three-dimensional shapes. Surface coatings can overcome most of the aforementioned limitations, but it has been recently demonstrated that any additional layer above the boiling surface increases overall thermal resistance that results in large temperature drops across the coating at high heat fluxes^25^. Therefore, future development for a technological market breakthrough in this field demands a straightforward, robust and scalable surface modification technique that will be able to enhance and control boiling heat transfer not only for water, but also for other types of (even low surface tension) fluids.

Here, we report on a fiber-laser texturing method that enables metal surface modifications by multi-level micro(μ)-cavities for enhanced and controlled saturated nucleate pool boiling performance for water as well as for small capillarity length tetradecafluorohexane (FC-72). The presented method overcomes the majority of the listed drawbacks since it avoids additional layer increasing overall thermal resistance, enables straightforward and robust production and significantly improves technical controllability of nucleate boiling, regardless of the capillarity distance and/or the wettability of the working fluid.

## Results and Discussion

### Laser texturing of multi-scale μ-cavities

Surfaces of 25 μm thick AISI 316 stainless steel foils were processed by direct laser texturing (DLT) using a nanosecond fiber laser (wavelength λ = 1060 nm) offering greater efficiency, reliability, low cost and the flexibility by ability to control the diversity of processing parameters^26^. The beam spot size with a diameter of 0.03 mm results in a pulse fluence of 8.5 J cm^−2^. Since this value is significantly higher than the threshold fluence for laser ablation, the laser-pulse-surface interaction leads to a surface crater surrounded by resolidified material^27^. The laser pulses were led over the surface by using a scanner with the velocity *v* = 0.15 m s^−1^. Thus, two successive pulses are separated by *Δx* = *v/f* = 6 μm inducing a channel of similar width as the beam spot diameter^28^.

**Figure 1:**
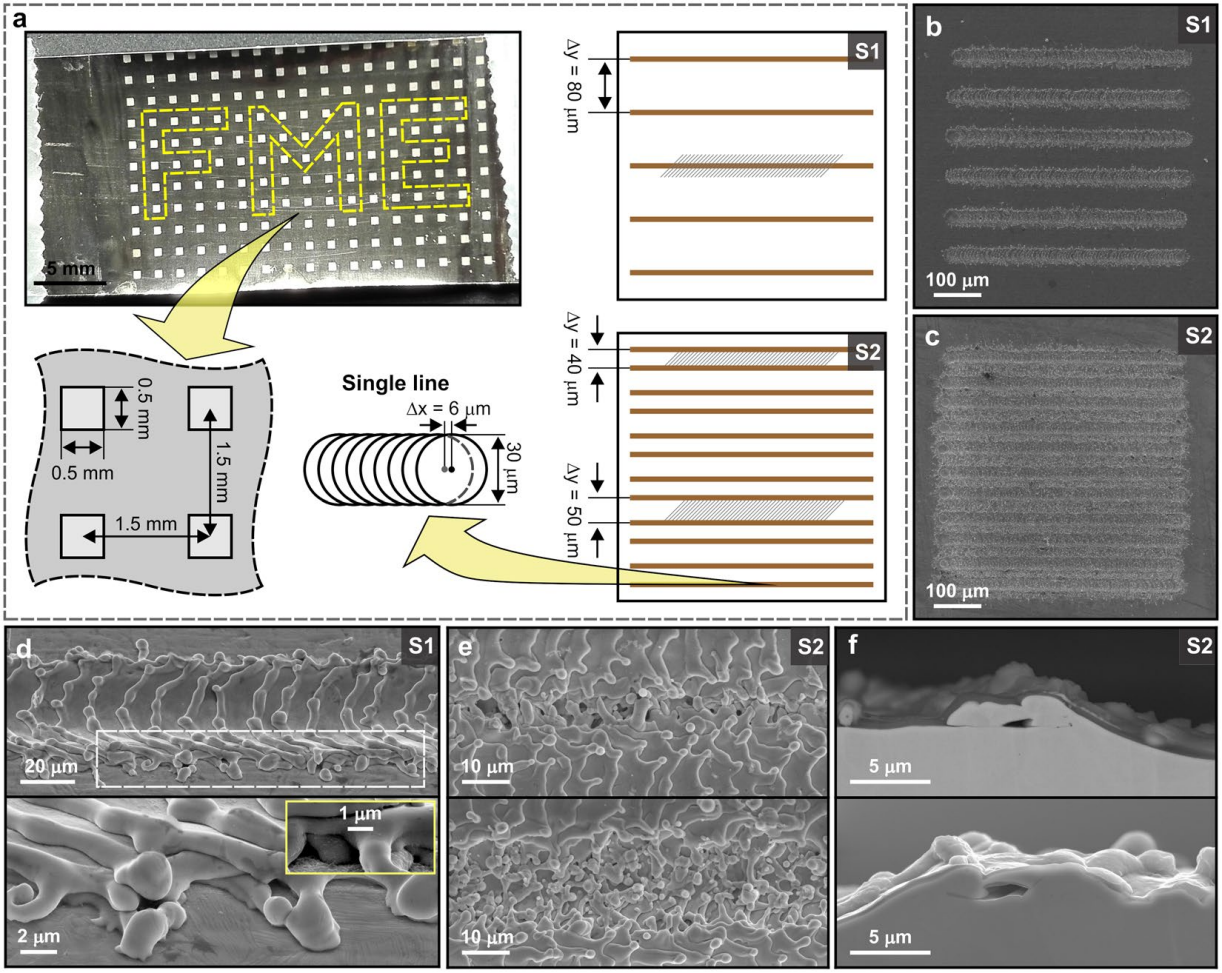
Laser-textured surfaces. (**a**) Stainless steel foil with laser textured array and schematic presentation of laser-texturing method. (**b**) SEM images of square, textured by S1 and (**c**) S2 structure. Magnified SEM images of (**d**) S1 structure [marked area in S1 in (**a**)] and (**e**) S2 structure in the border between two channels [marked areas in S2 in (**a**) – top for *Δy* = 40 μm, bottom for *Δy* = 50 μm]. (**f**) Cross section of S2.

Two types of surface structure, labelled S1 and S2, were prepared by changing the scanning line separation, as schematically shown in Fig. 1a. In the first case (S1), we created a texture with well-separated channels (*Δy* = 80 μm, Fig. 1b), while in S2 case the scanning line separation was alternated between the values *Δy* = {40 μm, 50 μm} (Fig. 1c). The scanning electron microscopy (SEM) micrographs of surface tilted for 60° of S1 channel reveal that μ-cavities are formed at the edges during the solidification of the remolten material after laser ablation (Fig. 1d, white dashed rectangle). SEM micrographs at higher magnifications clearly show that the diameter of these μ-cavities is within the range of 0.2–1.5 μm. The situation is slightly different for S2, where the scanning line separation is decreased to the values just slightly larger than the channel width. Here, the range of μ-cavities diameters is significantly increased due to overlapping of the resolidified material at the edges of the neighbouring channels (Fig. 1e) and equals to 0.2–10 μm. Moreover, the S2 structure becomes porous as the cavities are much deeper compared to S1 and some of them are also interconnected to each other, as revealed by a cross-section in Fig. 1f.

As confirmed by the results of the nucleate boiling experiments in the next subsection, multiscale μ-cavities that are interconnected to each other are of a crucial importance for the controlled and enhanced heat transfer. Our systematic examination of laser parameters (see Supplementary Figs 1 and 2) revealed that one of two possible strategies can be used to produce such surface structures by fiber-laser texturing in a robust and straightforward way. The first one is to use the right combination of (lower) pulse fluences and scanning line separation, as in case of the presented experiments. However, in this strategy only small variations in one of these two parameters can lead either to closed gap - without micro cavities due to too small distance between two consecutive μ-channels (Supplementary Fig. 1a) - or to too wide gap lacking appropriate μ-cavities (Supplementary Fig. 1b). The robustness of this approach can be significantly improved by using multiple scanning line separations, as in case of our experiments where we processed the surface by two different *Δy*. This strategy enables also to use lower costs scanners with lower angular (positioning) resolution. Moreover, such approach additionally leads to higher diversity of μ-cavities’ diameters and, thus, enable higher degree of enhancement of the boiling process.

The second strategy is to use higher pulse fluences leading to higher amount of the resolidified material around micro channels (Supplementary Fig. 2). However, this strategy is not appropriate for processing 25 μm thin foils that are required for measuring the transient temperature fields by an IR camera on the backside of the foil, as performed in our experiments. Nevertheless, it can be successfully employed in real production for modifications of different types of heat exchangers, where much thicker surfaces are used.

### Importance of multi-scale μ-cavities on boiling performance

To demonstrate the importance of multi-scale μ-cavities on boiling performance, two foils were laser textured, one by the S1 and the second by the S2 structure, as shown in Fig. 1a. Here, the surface structure is processed within 0.5 × 0.5 mm^2^ squares forming an equidistant array pattern with 1.5 mm pitch in both directions. Typical surface morphology of processed squares is revealed in Fig. 1b,c. Foils were tested for nucleate boiling enhancement.

The idea of the nucleate boiling enhancement can be, in general, described by two steps. Firstly, the temperature of the ONB should be minimized to increase the heat transfer coefficient at the very beginning of the nucleate boiling regime^12,29^. Secondly, as the heat flux increases, the nucleation frequency, nucleation site density and liquid suction towards the nucleation sites should also be increased, and the bubble coalescence should be prevented to delay the formation of large dry-out areas that might lead to the onset of CHF conditions^13,16,30,31^. According to the Hsu’s nucleation criterion the temperature of the ONB could be minimized, if the surface incorporated optimally sized vapour-trapping cavities^32,33^. Furthermore, to achieve a high probability for bubble nucleation at any given wall temperature (or heat flux), the cavities diameters (*d*_c_) should fall within a certain range^32^:

1 $$\{{d}_{{\rm{c}},\min },{d}_{{\rm{c}},\max }\}=\delta (\frac{\sin \,\theta }{1+\,\cos \,\theta })(1\mp \sqrt{1-\frac{8\sigma {T}_{{\rm{sat}}}(1+\,\cos \,\theta )}{{h}_{{\rm{lv}}}{\rho }_{{\rm{v}}}\delta ({T}_{{\rm{w}}}-{T}_{{\rm{sat}}})}})\,,$$

where the *θ*, *σ*, *h*_lv_ and *ρ*_v_ are the contact angle, surface tension of liquid with respect to its vapor, latent heat of vaporization and vapour density, respectively. Thermal boundary layer (*δ*) can be defined as the ratio between thermal conductivity of the liquid (*λ*_l_) and heat transfer coefficient for natural convection (*α*_*nc*_). *T*_w_ stands for the wall temperature of the boiling surface and *T*_sat_ is the saturation temperature of the fluid. Equation (1) is derived for saturated boiling conditions and is a simplified form of Hsu’s nucleation criterion, which generally applies to saturated and subcooled boiling.

To investigate the versatility of laser-textured surfaces, we used two very contrasting fluids for boiling experiments: water, which is polar, possess high surface tension and high latent heat of vaporization, and FC-72 as a non-polar, dielectric and low-surface-tension fluid with much lower latent heat (Supplementary Table 1). In our case, the contact angle of water on S2 equals to 16.3° ± 3.5° due to laser texturing^28,34^ and for FC-72 it equals to 12.7° ± 2.0° (Supplementary Fig. 3), and the wettability of the S1 is not significantly different as on S2.

Based on Equation (1) and considering pool-boiling operation at α_nc_ ≈ 3000 W m^−2^ K^−1^ and *T*_w_-*T*_sat_ ≈ 15 K, the active cavity diameter on the surface with a static contact angle of 15° should be roughly within the 0.3–60 µm range for water and the 0.03–5 µm range for FC-72 (Supplementary Fig. 4). Thus, the required μ-cavity diameter range for water is approximately one order of magnitude larger than for FC-72. This theory shows that boiling surface should include multi-scale μ-cavities in order to enhance the nucleation process for different types of fluids. The presented method using a nanosecond fiber laser is capable of producing such surfaces, as confirmed by Fig. 1e,f and discussed in previous subsection. Moreover, the Hsu’s nucleation criterion also show that not wettability itself, but the μ-cavities play a crucial role in boiling performance. The wettability just shifts the range of the required μ-cavities’ diameters^33^. This is an additional benefit of the proposed method, since when the enough wide range of the μ-cavities’ diameters is produced, the boiling performance become almost independent on the wettability, which additionally increases its robustness and overcame the drawbacks (including the change of the surface wettability after laser texturing^28,34–36^) of the existing methods for enhanced boiling process.

**Figure 2:**
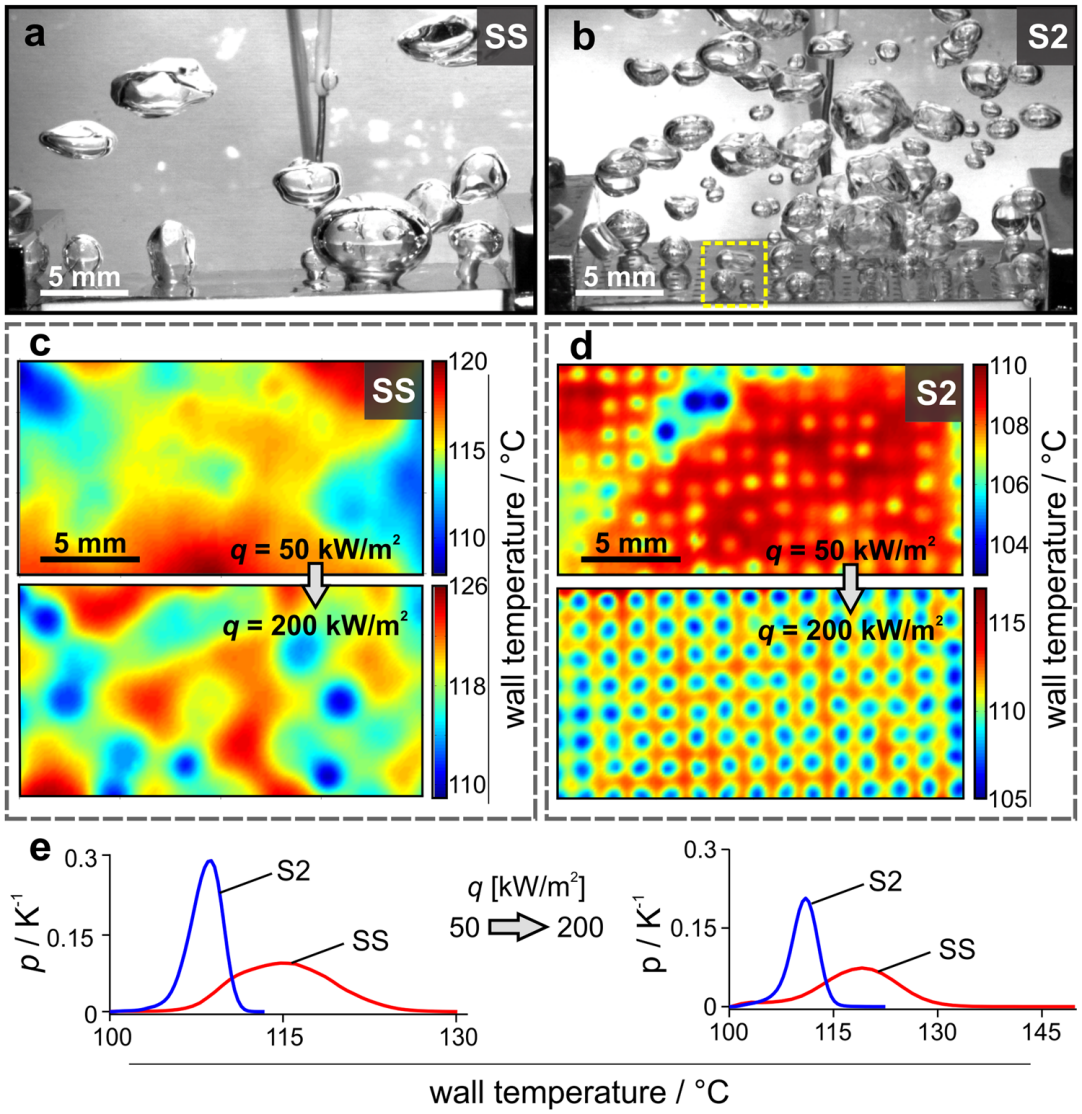
Saturated water nucleate pool boiling on as-received (SS) and laser textured foil with S2 structure. (**a**) Representative high-speed video image of boiling on SS and (**b**) S2 array at 50 kW m^−2^. (**c**) Time-averaged IR thermographs of SS and (**d**) S2 array at 50 kW m^−2^ (top) and 200 kW m^−2^ (bottom). (**e**) Wall-temperature distributions for SS and S2 array at 50 kW m^−2^ (left) and 200 kW m^−2^ (right).

The boiling performance was evaluated with high-speed visualization of nucleating bubbles and high-speed IR thermography measurements of the boiling surface transient temperature fields. As a reference surface, we used unprocessed (as-received, smooth) stainless steel foil (SS) with an average surface roughness *Sa* = 0.07 µm. The saturated pool boiling experiments using redistilled water (Fig. 2a) show that nucleation site density on reference surface is below 1 cm^−2^ at 50 kW m^−2^ and bubble departure diameters equal to 3.5 ± 1.4 mm (Supplementary Fig. 5). On the contrary, surface S2, covered by multi-scale cavities, provides bubble departure diameters of 2.8 ± 0.8 mm and nucleation site density of 44 cm^−2^ (Supplementary Fig. 6). Time-averaged IR thermographs on Fig. 2c,d reveal that nucleation sites, which are observed as cold spots, are randomly distributed on the SS sample and very deterministically distributed on the S2 surface. In the thermographs, every laser-textured square represents an active nucleation site. Relatively high surface temperature is required for bubble activation on the untreated surface due to the lack of vapour-trapping μ-cavities. High initial energy results in larger bubbles, larger bubble contact diameters and larger average wall temperature compared to the S2 surface. The wall temperature distribution for the SS sample at 50 kW m^−2^ (Fig. 2e) shows that local maximum wall temperature reached 130.4 °C with a high standard deviation of 4.1 K. Contrastingly, the S2 surface exhibits a maximum temperature of only 113.6 °C with a significantly lower standard deviation of 1.5 K.

Here, it should be noted that time-averaged IR thermographs (Fig. 2c,d) show *time-averaged* temperature fields at 50 kW/m^2^ and 200 kW/m^2^ for SS and S2 surfaces, respectively. They are calculated on 10 s of recording at 1,000 fps (e.g., from 10,000 frames). Therefore, each pixel in average temperature fields is calculated as an average of 10,000 temperature points. The main purpose of such data processing is to visualize locations of active nucleation sites (marked as blue/cold regions in average temperature field) and non-active boiling regions (marked as red/hotter areas). However, the *average* temperature field (Fig. 2c,d) is not an indicator of *local* maximum temperature that was measured in a certain point within the 10 s measuring span. Local maximum value is obtained from wall temperature distribution shown in Fig. 2e and it is, therefore, higher as the maximum temperature of the *time-averaged* temperature field, that can be identified from the colour-bar scale in Fig. 2c,d.

As the heat flux increases, the differences between SS and S2 sample become even more pronounced. At 200 kW m^−2^ the nucleation site density on SS equals 6 cm^−2^ and on S2 sample 44 cm^−2^. High number of equally distributed active nucleation sites on laser-textured surface provide heat transfer coefficient of 24.4 kW m^−2^ K^−1^ and wall temperature deviation of 2.2 K. This is significant enhancement compared to bare stainless steel, where we measured heat transfer coefficient of 12.2 kW m^−2^ K^−1^ and wall temperature deviation of 5.8 K. This leads to a conclusion that the laser-textured array of squares covered by multi-scale μ-cavities enhances boiling incipience for water, reduces local maximum and average wall temperature (e.g., increases the heat transfer coefficient) and provides deterministic and stable boiling behaviour, since the bubble contact diameters and wall temperatures are in a very narrow range compared to the untreated surface.

### Boiling controllability in different fluids

**Figure 3:**
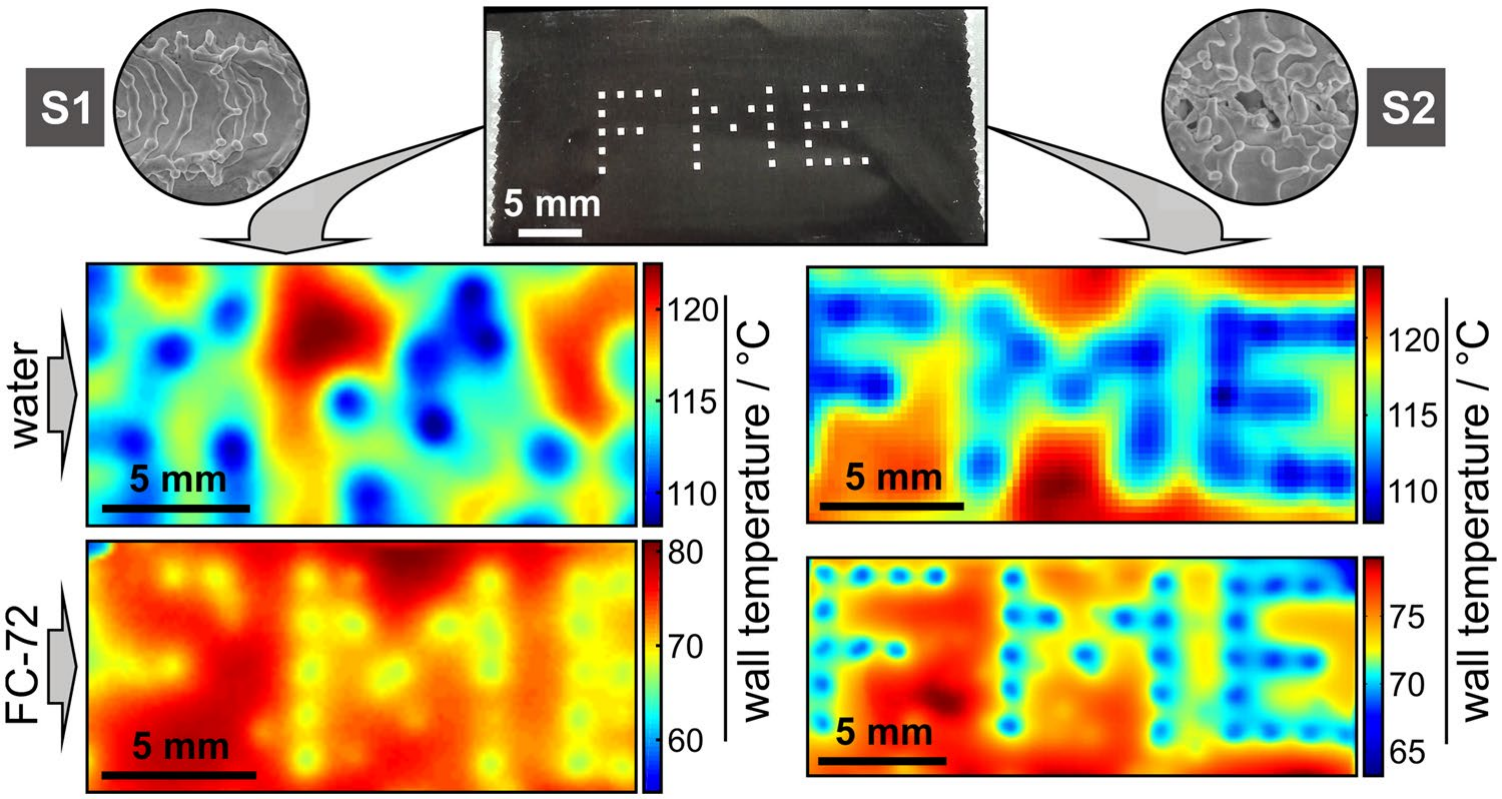
Controlled nucleate boiling performance of water and FC-72. Photography of laser-textured “FME” pattern of S1 (left) and S2 (right) structure is shown on the top. Average temperature fields of boiling surfaces are presented for water at 150 kW m^−2^ (top) and FC-72 at 15 kW m^−2^ (bottom).

To demonstrate the capability of the developed fiber-laser texturing method for the enhancement and high degree of control over the boiling process within different types of fluids, we prepared surfaces with an “FME” (Faculty of Mechanical Engineering) pattern (selected squares, highlighted by the yellow line in Fig. 1a). Here, structures S1 and S2 were used to demonstrate the importance of a wide range of μ-cavity diameters. The distribution of these diameters within S1 (0.2–1.5 µm) is not optimal for water according to the Hsu’s nucleation criteria, but falls within the desired range for FC-72 and other low-surface-tension fluids. Time-averaged wall temperature images during boiling indeed confirm that S1 structures do not present active nucleation sites for water, but they are effective for FC-72 (left-hand side of Fig. 3). However, the S2 structure, which provides a one order of magnitude larger range of μ-cavity diameters (0.2–10 µm), enhances boiling heat transfer for both water and FC-72. The “FME” structure is clearly visible as a cooled-down region due to constant boiling activity and provides on average 11 K (for water at 150 kW m^−2^) and 7 K (for FC-72 at 15 kW m^−2^) lower surface temperature compared to the surrounding inactive regions.

### Demonstration of controlled nucleate boiling performance of ethanol and overlook

In previous subsections, we have successfully demonstrated that the developed fiber-laser-texturing method enable roust and straightforward production of the surface for enhanced and controlled heat transfer by nucleate boiling without any additional surface coatings. This was proved by a detailed testing of the developed S2 surface in two very contrasting fluids. One is water as a polar fluid with extremely high surface tension and extremely high heat of vaporization. On the other hand, we have chosen also non-polar, dielectric FC-72 that has among the lowest surface tension of all fluids we currently know. Based on this criteria most of the pure fluids currently used in phase-change heat transfer applications fall within water and FC-72.

The experiments with water were performed in the range of 25–300 kW m^−2^ (by step of 25 kW m^−2^), while boiling of the FC-72 we analyzed within the range of 5–90 kW m^−2^ (by step of 5 kW m^−2^). Nevertheless, it should be mentioned that the quantitative comparison of transient temperature fields between water and FC-72 are very limited by the current experimental setup due to significantly different latent heat of both fluids. When water is used, each nucleating bubble removes relatively large amount of heat due to high latent heat of the water (e.g., 2,257 kJ kg^−1^). This results in significant local temperature drop that is transferred to the bottom site of the foil, and measured with a high-speed IR camera with expanded measurement uncertainty of ±2 K. On the contrarily, the FC-72 has the latent heat of only 88 kJ kg^−1^; thus, the local heat fluxes due to bubble growth are more than one order of magnitude lower compared to water. As a result, the temperature variations observed on the bottom side of the foil are significantly affected by the limited thermal response of 25-µm foil. This is the main reason that we were able to show quantitative experimental results for water (Fig. 2), but only qualitative comparison between water and FC-72 (Fig. 3 and Supplementary Figs 5,6). Since water and FC-72 are completely different fluids, they generally require different experimental setups. However, in evaluating of boiling performance, the quantitative comparison of different working fluids, such as the nucleation frequency and the active nucleation site density are of great importance^33^. In the future work the current experimental setup should be improved in sense of using even thinner titanium foils that will enable such an analysis of the boiling performance of FC-72 on laser textured surfaces.

**Figure 4:**
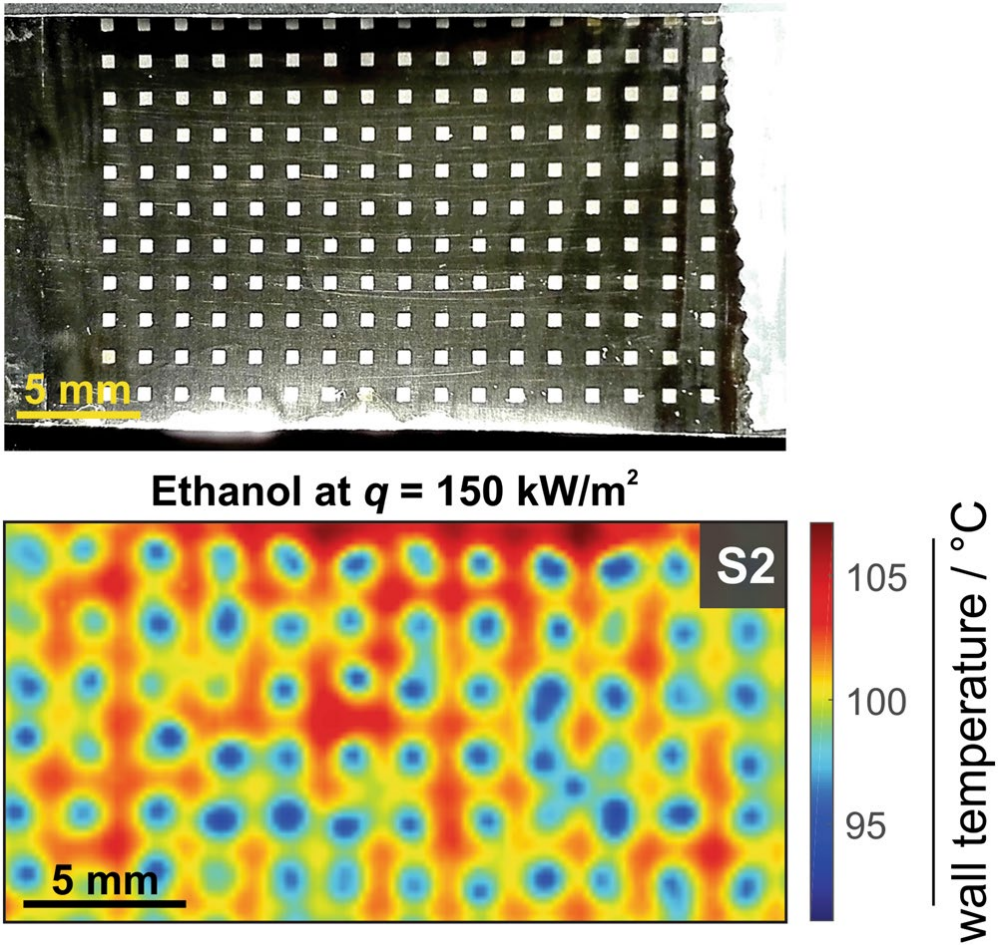
Controlled nucleate boiling performance of ethanol. Photography of laser-textured pattern of S2 structure (top) and average temperature fields of saturated nucleate boiling of ethanol at 150 kW m^−2^ (bottom).

Additional important challenge that should be addressed in the future work is testing of the developed surfaces in other fluids whose properties fall within water and FC-72, and are used in variety of phase-change applications. This will lead to important breakthrough needed to overcome the current limitations for further miniaturization of microelectronic devices and to increase performance and safety of different heat flux systems. To additionally prove, that our method has the ability to address these challenges and that further investigation by using different, improved experimental setups and different fluids (including fluid mixtures), is worth to perform, we tested the developed S2 surface on boiling performance of an ethanol (its properties are listed in Supplementary Table 1). Here, we used the squares (by a same pattern as sketched in Fig. 1a) that were covered by S2 structure. The obtained results are presented in Fig. 4.

Time averaged temperature field at 150 kW m^−2^ show that also for ethanol each laser textured square region represents active nucleation site, creating equally distributed nucleation pattern across entire boiling surface. In this case, we measured average wall temperature on active nucleation sites to be 8.4 K lower compared to surrounding non-active boiling area. Local time-averaged heat transfer coefficient on nucleation sites was 9.9 kW m^−2^ K^−1^, but on non-active area in between nucleation sites only 6.3 kW m^−2^ K^−1^. These results additionally confirm that the surfaces, covered by multiscale μ-cavities enable a general approach for enhanced boiling performance, almost independently on different fluid properties, including their surface tension.

## Conclusions

We have demonstrated that surface texturing by a nanosecond fiber laser enables straightforward, compact, robust, flexible, chemical-free and cost–effective method with fast processing times for producing structured metal surfaces with multi-level μ-cavities. Additionally, this method avoids additional surface layer, as in case of surface modification by different coatings and, consequently, such laser-textured surfaces does not increase overall thermal resistance. The presented results, therefore, confirm that the developed laser processing method overcomes the majority of the drawbacks of currently available approaches for engineering the surfaces for enhanced and controlled boiling heat transfer. Our results also confirm the Hsu’s criterion and reveal that such surfaces present a general approach to enhanced heat transfer by controlled boiling in a diverse range of fluids, independently on their polarity or surface tension. This opens new possibilities for overcoming the limitations for further miniaturization of microelectronic devices, and has a high potential to increase the performance and safety in high heat flux systems.

## Methods

### Material and surface characterization

25-µm thick stainless steel foils (S316, Precision brand) of 27 × 17 mm^2^ (Fig. 1a) were used for all experiments. Chemical composition was determined using X-ray fluorescence spectrometer (XRF, Thermo Scinetific Niton XL3t GOLDD+) and carbon and sulphur analyser (ELTRA CS-800): 0.40% Si, 1.53% Mn, 16.0% Cr, 10.5% Ni, 0.34% Cu, 1.46% Mo, 0.13% V, 0.030% C, <0.005% S and Fe rest in mass fraction. SEM images were recorded using JEOL JSM-6500F at 15 kV. Cross section of S2 was performed by JEOL SM-09010 polisher. Surface roughness was measured using 3D optical interferometric microscope Bruker Contour GT-K0.

### Laser texturing

Stainless steel foils were textured by a fiber laser (SPI Lasers, Ltd., G4, SP-020P-A-HS-S-A-Y), radiating pulses with a wavelength of 1060 nm, an average power of 1.5 W, and pulse duration of 28 ns at full width at half maximum and 50 ns at 10% of the peak power. The beam with a diameter of 0.03 mm was lead across the surface in lines by a scanner (Raylase, SS-IIE-10) with the velocity of 150 mm s^−1^; the pulse frequency equalled 25 kHz. On the S1 sample, the lines were separated by *Δy* = 80 μm, while for the S2, this distance was alternated between the values *Δy* = {40 μm, 50 μm}. Three foils were textured by fiber laser: the first by array of equally separated squares (0.5 × 0.5 mm^2^, pitch of 1.5 mm, Fig. 1a) filled with S2. Two additional foils had “FME” pattern (Fig. 3) made of same squares – one filled with S1 and the second with S2 structure.

### Boiling experiments

The experimental setup for the saturated pool-boiling experiments is similar as in our previous studies^12,33^. It uses a 170 × 100 × 100 mm^3^ pool-boiling chamber. The working fluids are degassed double-distilled water (Roth 3478.2) and 1,1,1,2,2,3,3,4,4,5,5,6,6,6-tetradecafluorohexane (FC-72, 3M), respectively. They condense on the top and return to the boiling chamber maintaining a surface at approximately 3.5 cm above the boiling surface. Immersed cartridge heaters are used for preheating, degassing and maintaining the saturation conditions (100 °C at 1 atm). Bulk temperature was measured with two thermocouples of type K. The ceramic base as a heater unit has two electrical contacts to hold and power the foil. The foil is heated using the Joule effect: heat flux equals the product of the current multiplied by the voltage drop across the foil, divided by its surface area. A constant heat generation across the foil is assumed. A borehole (23 × 13 mm^2^) milled in the base’s centre enables recording of the transient temperature fields on foil’s underside. A golden mirror placed below the heater (at 45°) reflects the thermal radiation to the IR camera (FLIR SC6000) which uses a frame rate of 1000 fps and a spatial resolution of 250 and 100 μm pixel^−1^ for water and FC-72 experiments, respectively. Simultaneously, synchronized high-speed video camera (Photron UX100 Mini) records the boiling process on the top of the foil.

The conversion from a raw digitalized IR signal to a temperature was calculated using a calibration curve obtained under the same ambient conditions as during the boiling experiments. The expanded absolute uncertainty of the temperature measurement was determined to be 2 K and was practically constant across the entire calibration range. However, the noise equivalent differential temperature (i.e., sensitivity) of the IR sensor was 20 mK. The uncertainty of the temperature difference among the individual pixels is therefore much lower than the absolute temperature uncertainty. The expanded relative measurement uncertainty of the heat flux was calculated to be 0.5%.

### Data Availability

The datasets generated during and/or analysed during the current study are available from the corresponding author on reasonable request.

## Electronic supplementary material

Supplementary Information
