# Supplementary material for: Scalable Surface Microstructuring by a Fiber Laser for Controlled Nucleate Boiling Performance of High- and Low-Surface-Tension Fluids
Source: Sci Rep. 2018 May 10;8:7461. doi: 10.1038/s41598-018-25843-5 (PMC5945849; doi:10.1038/s41598-018-25843-5)
Supplement: Supplementary file 1 — Supplementary Information [file 41598_2018_25843_MOESM1_ESM.docx]

Supplementary Information

Scalable Surface Microstructuring by a Fiber Laser for Controlled Nucleate Boiling Performance of High- and Low-Surface-Tension Fluids

Peter Gregorčič*, Matevž Zupančič, Iztok Golobič

*University of Ljubljana, Faculty of Mechanical Engineering, Aškerčeva 6, 1000 Ljubljana, Slovenia*

**Supplementary Figures**


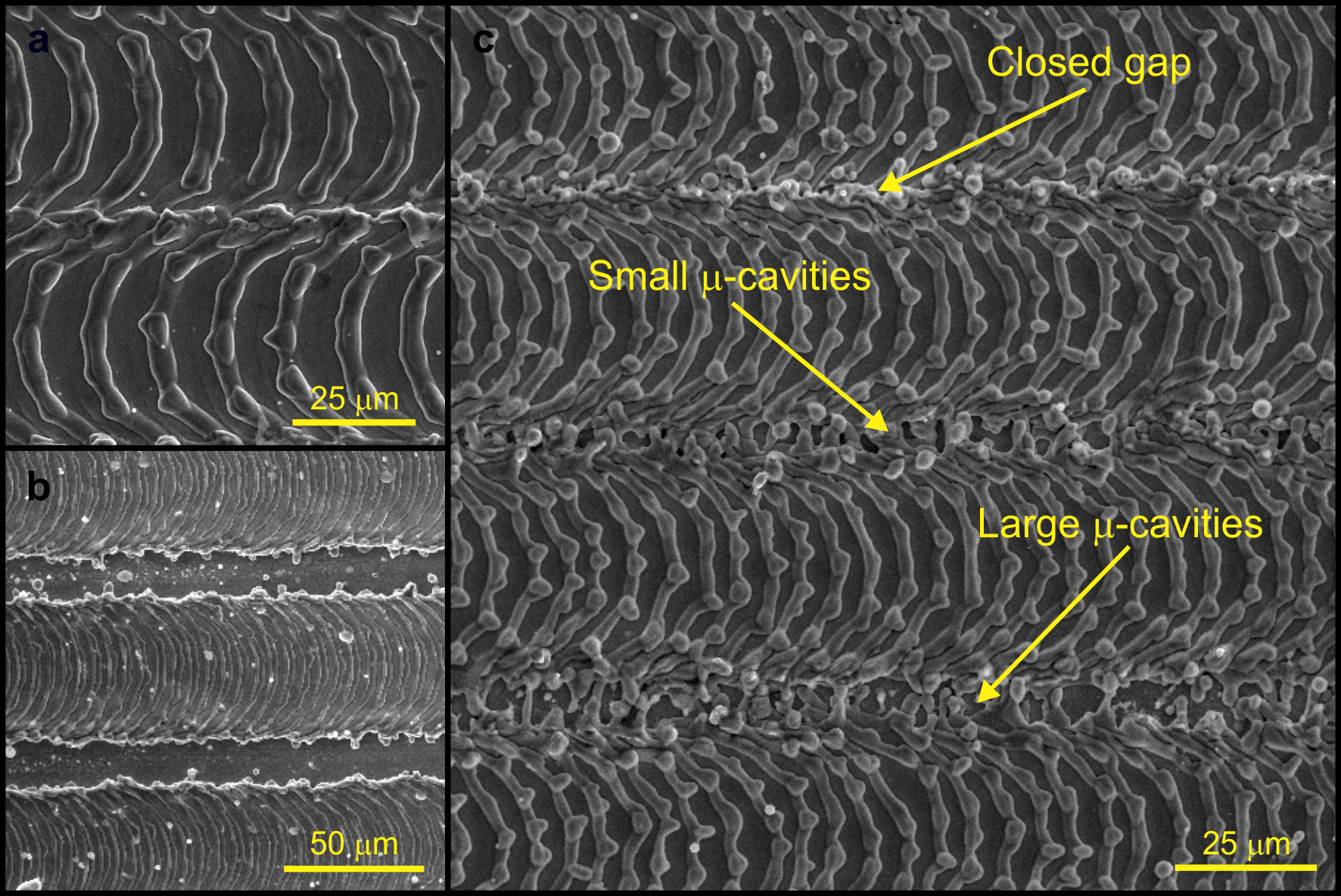


**Supplementary Figure 1.** Laser texturing of thin (25 μm) foils requires appropriately low pulse fluences. In this case, the right combination of pulse fluences and scanning line separation should be used to obtain μ-cavities within the border between two neighbouring μ-channels. Here, only small variations in one of these two parameters can lead either to **(a)** closed gap - without micro cavities due to too small distance between two consecutive micro channels (Δ*y* = 35 μm, pulse fluence of 6.9 J cm^-2^) - or **(b)** to too wide gap lacking appropriate μ-cavities (Δ*y* = 65 μm, pulse fluence of 6.9 J cm^-2^ ). **c,** The robustness of this approach can be significantly improved by using multiple scanning line separations (Δ*y* = {35, 40, 45} μm, pulse fluence of 8.5 J cm^-2^). In this case not only robustness of the processing is enhanced, but this also leads to higher diversity of μ-cavities’ diameters and, thus, enable higher degree of enhancement of the boiling process.


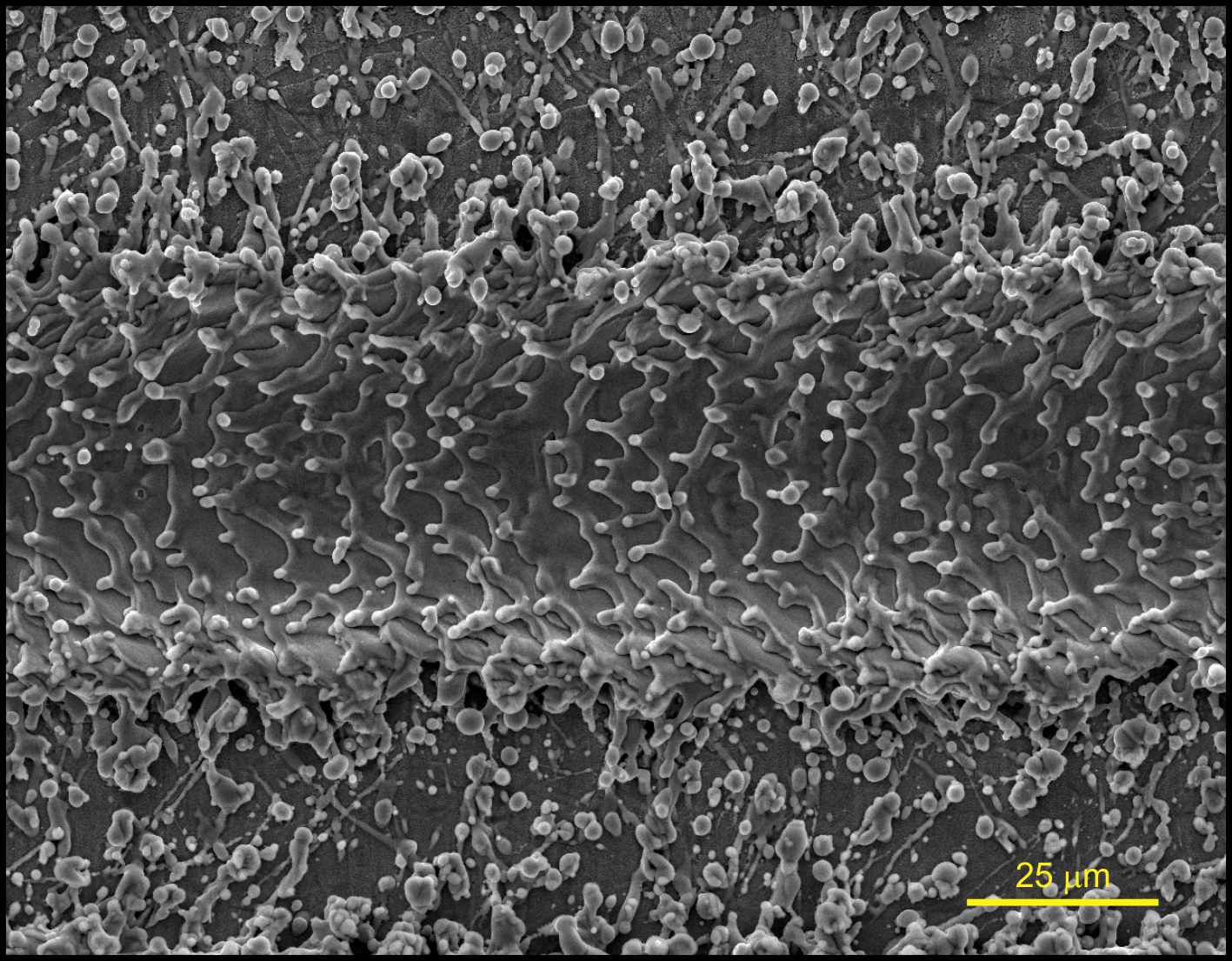


**Supplementary Figure 2.** The multi-scale μ-cavities can be obtained also by using higher fluences (in this case fluence equals 26 J cm^-2^). Here, the ejection of the remolten material itself leads to appropriate μ-cavities and combination of appropriate scanning line separation can only additionally enhance this approach in context of surface functionalization for enhanced boiling process. However, this approach is – due to too high fluences – not appropriate for processing thin (25 μm) foils, but it can be used in real production for modifications of different types of heat exchangers, where surfaces are significantly thicker.


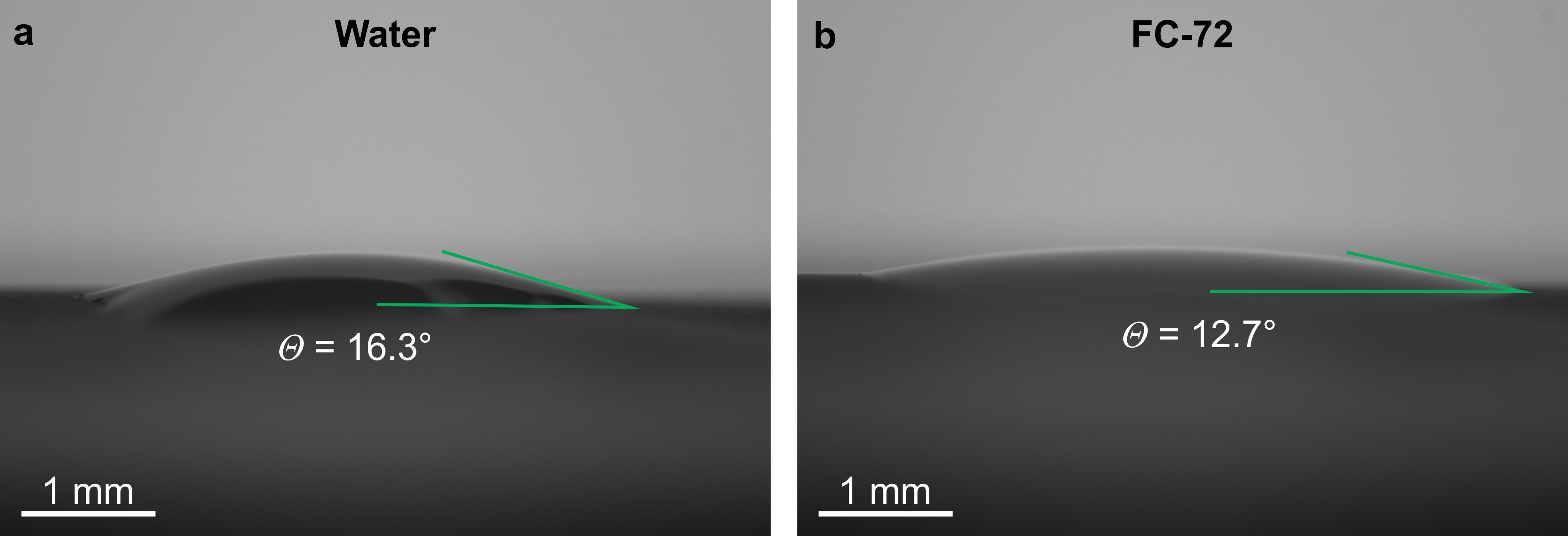


**Supplementary Figure 3. a,**  static contact angles on S2 structure for water and **b,** FC-72. For the contact angle measurements, we structured the entire 25 µm stainless steel foil (27 × 17 mm^2^) with laser scan line separation of *Δy* = [40 μm, 50 μm] to produce the S2 structure. Contact angles were measured at 25 °C with Krüss DSA 100. For each fluid we repeated the measurements 10 times on different parts of the surface and it was found that water contact angle on S2 structure equals 16.3° ± 3.5° and for FC-72 it is 12.7° ± 2.0°. The measurements were performed 1 week after laser texturing and immediately before the boiling experiments.


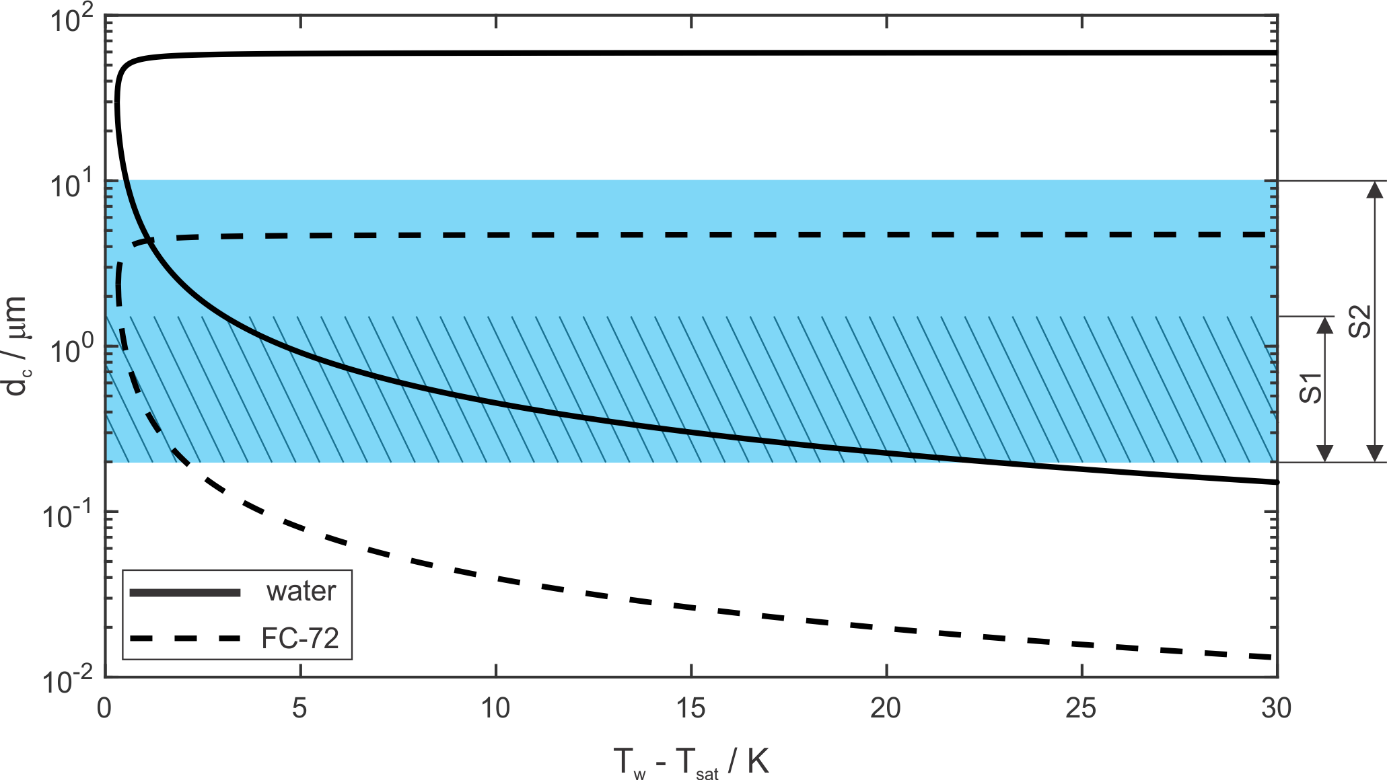


**Supplementary Figure 4.** Effective μ-cavity diameter range (*d*_c_) versus wall superheat (*T*_w_ – *T*_sat_) for water and FC-72 calculated from Hsu’s nucleation criterion. The calculation considers saturated boiling at atmospheric pressure and a static contact angle of 15°. Actual μ-cavity diameter ranges for S1 and S2 structures are marked on the right-hand side of the graph. It is visible that S2 surface provides a wider range of μ-cavity sizes covering larger theoretical range of active nucleation sites for both fluids.


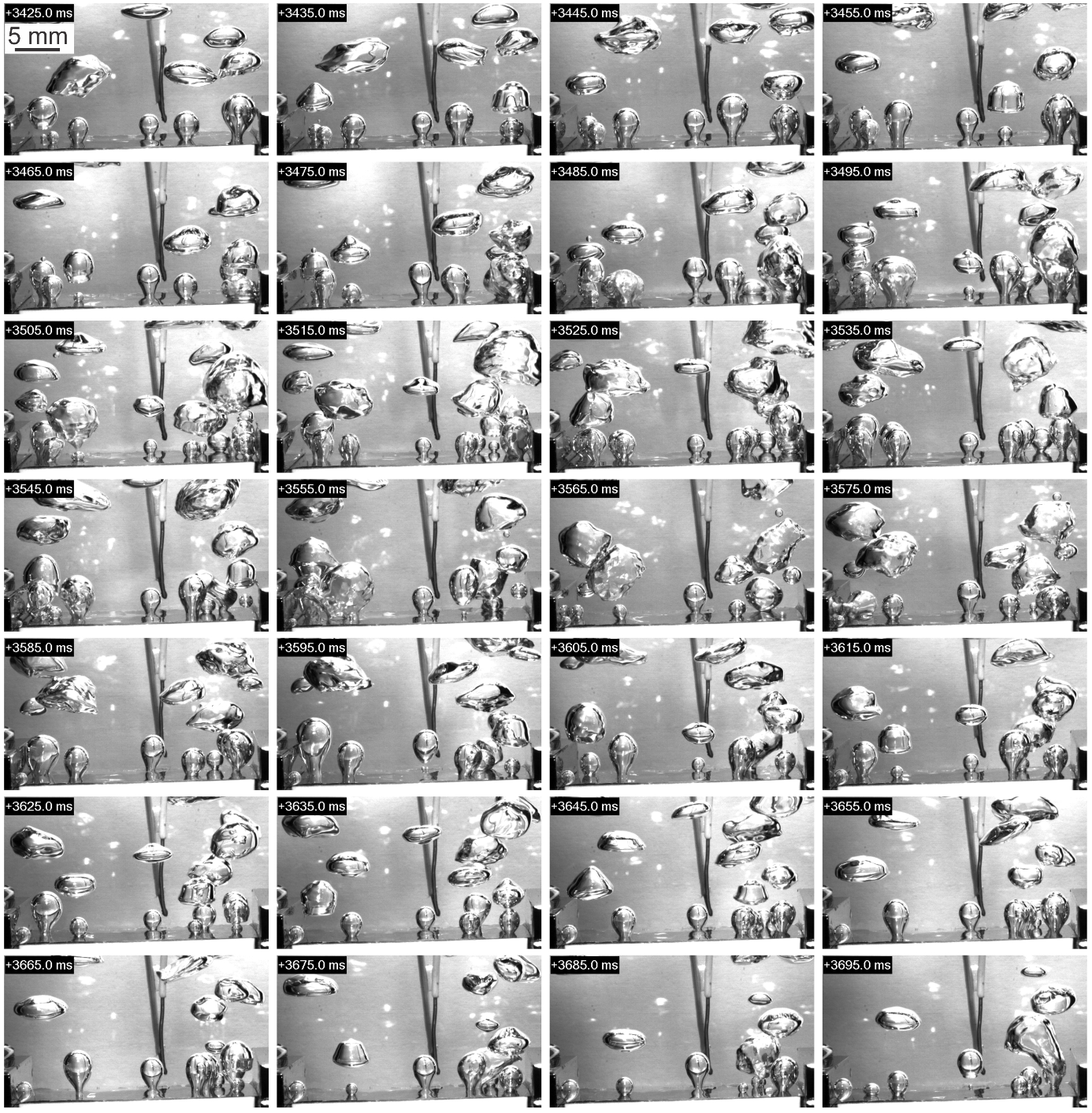


**Supplementary Figure 5.** Consecutive video images of saturated nucleate boiling of water on non-processed stainless steel foil (SS) at 50 kW m^-2^. Time step between images is 10 ms.

Figures S3 and S4 are showing selected successive images of saturated nucleate boiling process of water at 50 kW m^-2^ for unprocessed (SS) and laser-textured (S2) stainless steel foils, respectively. It is shown that array of S2 structures provides significantly higher nucleation site density, a higher nucleation frequency and smaller bubbles, compared to SS surface.


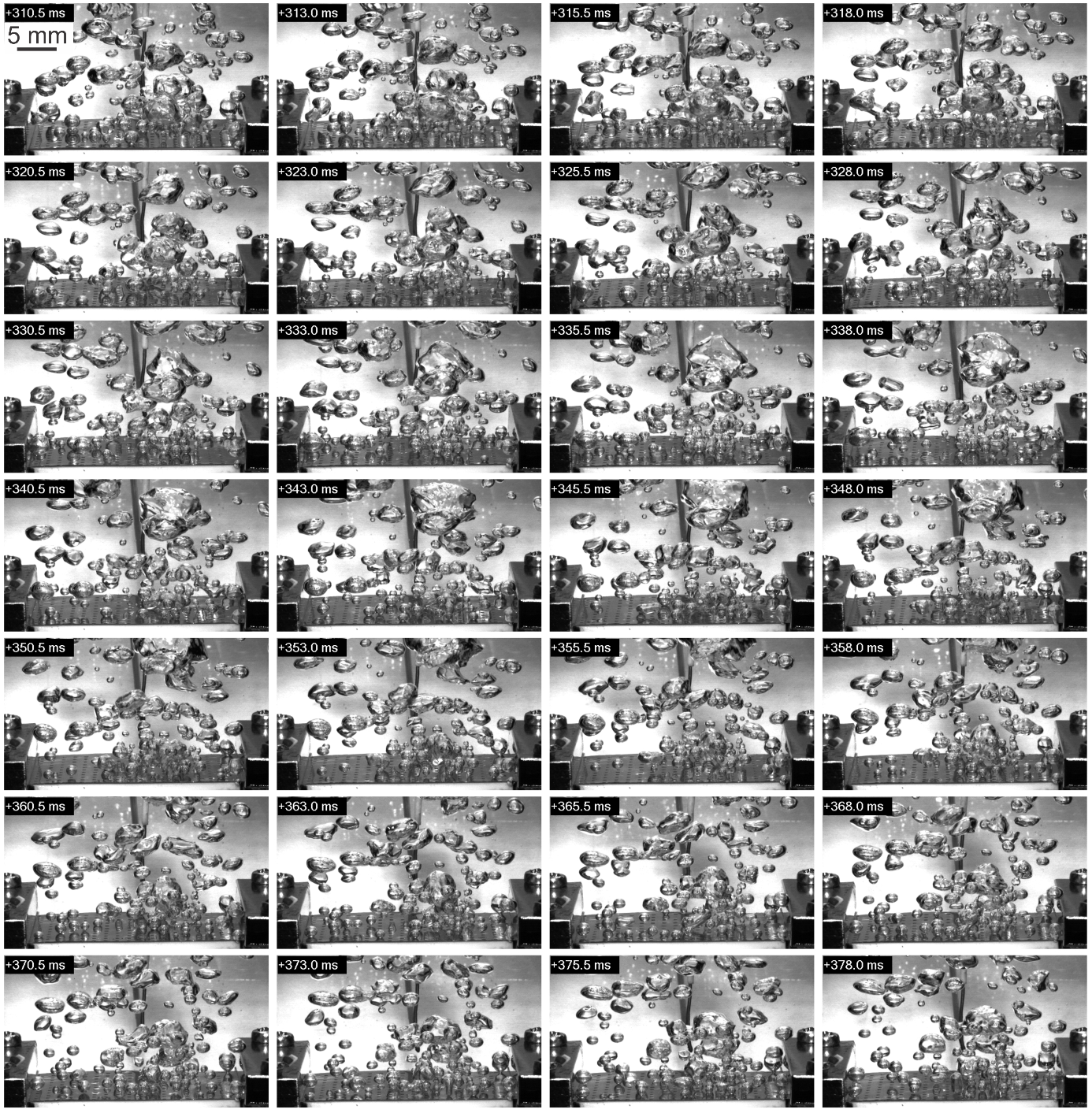


**Supplementary Figure 6.** Consecutive video images of saturated nucleate boiling of water on laser-textured stainless steel foil (S2) at 50 kW m^-2^. Time step between images is 2.5 ms.

**Supplementary Table**

**Supplementary Table 1.** Physical properties of water, FC-72 and ethanol at saturation conditions. These properties are needed for the calculation of μ-cavity diameter range according to Hsu’s nucleation criterion described by Equation (1).

| Fluid type^a)^ | *Saturation temperature, T_sat_*  *[°C]* | Surface tension, *σ*  *[mN m^-1^]* | Latent heat of vaporization, h_lv_  [kJ kg^-1^] | Vapor density, ρ_v_  [kg m^-3^] | Thermal conductivity of liquid, *λ*_l_  [W m^-1^ K^-1^] |
| --- | --- | --- | --- | --- | --- |
| Water (Roth CAS 7732-18-5) | 100 | 58.9 | 2257 | 0.60 | 0.679 |
| FC-72 (3M Fluorinert FC-72) | 56 | 8.0 | 88 | 13.43 | 0.054 |
| Ethanol (Merck CAS 64-17-5) | 78.4 | 17.4 | 850 | 1.65 | 0.154 |

^a)^ Fluid properties are given at 1 atm and saturation temperature conditions.
